# Renoprotective effects of empagliflozin in high-fat diet-induced obesity-related glomerulopathy by regulation of gut-kidney axis

**DOI:** 10.1152/ajpcell.00367.2024

**Published:** 2024-08-26

**Authors:** Lei Lei, Ting Zhu, Tian-Jiao Cui, Yvonne Liu, Johann-Georg Hocher, Xin Chen, Xue-Mei Zhang, Kai-Wen Cai, Zi-Yan Deng, Xiao-Hua Wang, Chun Tang, Lian Lin, Christoph Reichetzeder, Zhi-Hua Zheng, Berthold Hocher, Yong-Ping Lu

**Affiliations:** ^1^Department of Nephrology, Center of Kidney and Urology, The Seventh Affiliated Hospital, Sun Yat-sen University, Shenzhen, People’s Republic of China; ^2^Fifth Department of Medicine (Nephrology/Endocrinology/Rheumatology), University Medical Centre Mannheim, University of Heidelberg, Mannheim, Germany; ^3^Medical Faculty of Charité Universitätsmedizin Berlin, Berlin, Germany; ^4^Institute for Clinical Research and Systems Medicine, Health and Medical University, Potsdam, Germany; ^5^Institute of Medical Diagnostics, IMD, Berlin, Germany; ^6^Clinical Research Center for Reproduction and Genetics in Hunan Province, Reproductive and Genetic Hospital of CITIC-XIANGYA, Changsha, People’s Republic of China; ^7^NHC Key Laboratory of Human Stem Cell and Reproductive Engineering, School of Basic Medical Science, Institute of Reproductive and Stem Cell Engineering, Central South University, Changsha, People’s Republic of China; ^8^Department of Nephrology, the First Affiliated Hospital of Jinan University, Guangzhou, People’s Republic of China

**Keywords:** empagliflozin, gut-kidney axis, lipid metabolism, microbiota, obesity-related glomerulopathy

## Abstract

The increasing prevalence of obesity-related glomerulopathy (ORG) poses a significant threat to public health. Sodium-glucose cotransporter-2 (SGLT2) inhibitors effectively reduce body weight and total fat mass in individuals with obesity and halt the progression of ORG. However, the underlying mechanisms of their reno-protective effects in ORG remain unclear. We established a high-fat diet-induced ORG model using C57BL/6J mice, which were divided into three groups: normal chow diet (NCD group), high-fat diet (HFD) mice treated with placebo (ORG group), and HFD mice treated with empagliflozin (EMPA group). We conducted 16S ribosomal RNA gene sequencing of feces and analyzed metabolites from kidney, feces, liver, and serum samples. ORG mice showed increased urinary albumin creatinine ratio, cholesterol, triglyceride levels, and glomerular diameter compared with NCD mice (all *P* < 0.05). EMPA treatment significantly alleviated these parameters (all *P* < 0.05). Multitissue metabolomics analysis revealed lipid metabolic reprogramming in ORG mice, which was significantly altered by EMPA treatment. MetOrigin analysis showed a close association between EMPA-related lipid metabolic pathways and gut microbiota alterations, characterized by reduced abundances of *Firmicutes* and *Desulfovibrio* and increased abundance of *Akkermansia* (all *P* < 0.05). The metabolic homeostasis of ORG mice, especially in lipid metabolism, was disrupted and closely associated with gut microbiota alterations, contributing to the progression of ORG. EMPA treatment improved kidney function and morphology by regulating lipid metabolism through the gut-kidney axis, highlighting a novel therapeutic approach for ORG.

**NEW & NOTEWORTHY** Our study uncovered that empagliflozin (EMPA) potentially protects renal function and morphology in obesity-related glomerulopathy (ORG) mice by regulating the gut-kidney axis. EMPA’s reno-protective effects in ORG mice are associated with the lipid metabolism, especially in glycerophospholipid metabolism and the pantothenate/CoA synthesis pathways. EMPA’s modulation of gut microbiota appears to be pivotal in suppressing glycerol 3-phosphate and CoA synthesis. The insights into gut microbiota-host metabolic interactions offer a novel therapeutic approach for ORG.

## Graphical abstract

**Figure:**
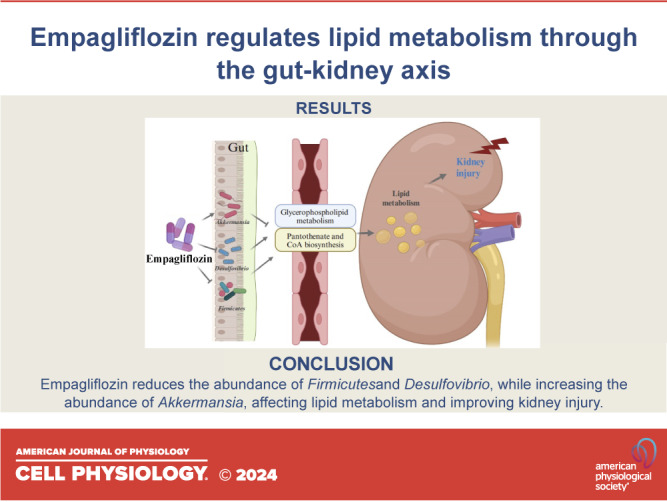

## INTRODUCTION

Obesity, a chronic metabolic disorder characterized by the accumulation of excessive body fat and associated with various health problems, contributes to the development of obesity-related glomerulopathy (ORG) (1). With the global prevalence of obesity, the incidence of ORG has significantly increased (2–4). ORG is an insidious-onset disease; up to one-third of patients with ORG may progress to chronic kidney disease (CKD) or even end-stage renal disease (ESRD) (3). The treatment for ORG is primarily based on weight reduction, supplemented by interventions such as blood pressure control, improvement of insulin resistance, and lipid-lowering (5–7). However, the exact pathogenic mechanism of ORG remains unclear, and targeted therapeutic approaches are limited. There is an urgent need to develop new treatment strategies to delay the progression of ORG.

ORG is a global health problem, and metabolic imbalance plays a pivotal role in its occurrence and progression (8, 9). Metabolites are intermediate and end products of the body’s biochemical reactions, providing accurate reflections of the metabolic characteristics of tissues and organs in physiological and pathological states. Metabolites are generated not only by the host organism but also by the gut microbiota, including short-chain fatty acids (10) and uremic toxins (11). In recent years, the application of sequencing technologies, such as metagenomics, in gut microbiota research has uncovered a close association between the gut microbiome and obesity. The findings emphasize that gut dysbiosis, impaired intestinal barrier function, and bacterial translocation are critical regulatory factors in the development of ORG (12). Currently, the molecular mechanisms underlying the regulation of ORG by the gut microbiota and its metabolites are not fully elucidated. The pathological process of ORG is complex and influenced by multiple factors. Identifying microbial metabolites originating from the gut that are associated with ORG and exploring the impact of the gut microbiota and its metabolites on kidney metabolism can contribute to a precise understanding of the pathogenic mechanisms of ORG. This, in turn, provides a theoretical basis for the intestinal treatment of ORG.

Sodium glucose cotransporter 2 (SGLT2) inhibitors, originally developed for the treatment of type 2 diabetes mellitus (T2DM), are clinically very effective drugs halting chronic kidney disease (CKD) progression (13). Recent studies have demonstrated that SGLT2 inhibitors exert both direct and indirect effects, reducing the risk of cardiovascular mortality and providing renal protective effects in patients with T2DM (14–16). These effects are independent of their glucose control effects but are associated with their impact on blood pressure control, amelioration of glomerular hemodynamics, regulation of the renin-angiotensin-aldosterone system (RAAS), anti-inflammation, and reduction of glucotoxicity, lipotoxicity, and uric acid. Furthermore, SGLT2 inhibitors are effective in inducing weight loss and reducing overall fat mass (17, 18). Empagliflozin (EMPA), a member of the SGLT2i inhibitors, has demonstrated a promising effect on reducing renal oxidative stress and inflammation in obesity (17, 19), as well as delaying the progression of obesity-related kidney dysfunction (20). Our previous studies have demonstrated that the renoprotective effects of empagliflozin in nondiabetic chronic kidney disease (CKD) are mediated through dose-dependent activation of the tubuloglomerular feedback mechanism and modulation of the complement system (21). Furthermore, empagliflozin has been shown to suppress macrophage-mediated renal inflammation and fibrosis (22). Furthermore, research suggests that EMPA can reshape the gut microbiota, leading to improved insulin sensitivity and blood glucose control (23), ameliorating atherosclerosis and systemic inflammatory response (24). Our previous study confirmed the impact of EMPA on kidney metabolism programming in diabetic kidney disease (25). However, the potential underlying mechanisms of the renoprotective effects of SGLT2 inhibitors in ORG are unclear.

In this study, we conducted 16S ribosomal RNA gene sequencing of feces and liquid chromatography-tandem mass spectrometry (LC-MS/MS)-based metabolomic analysis of kidney, feces, liver, and serum samples in ORG mice. We aimed to establish the gut-kidney axis to elucidate the underlying mechanisms of the renal protective effects of EMPA in ORG.

## METHODS

### ORG Model

**Figure 1.**
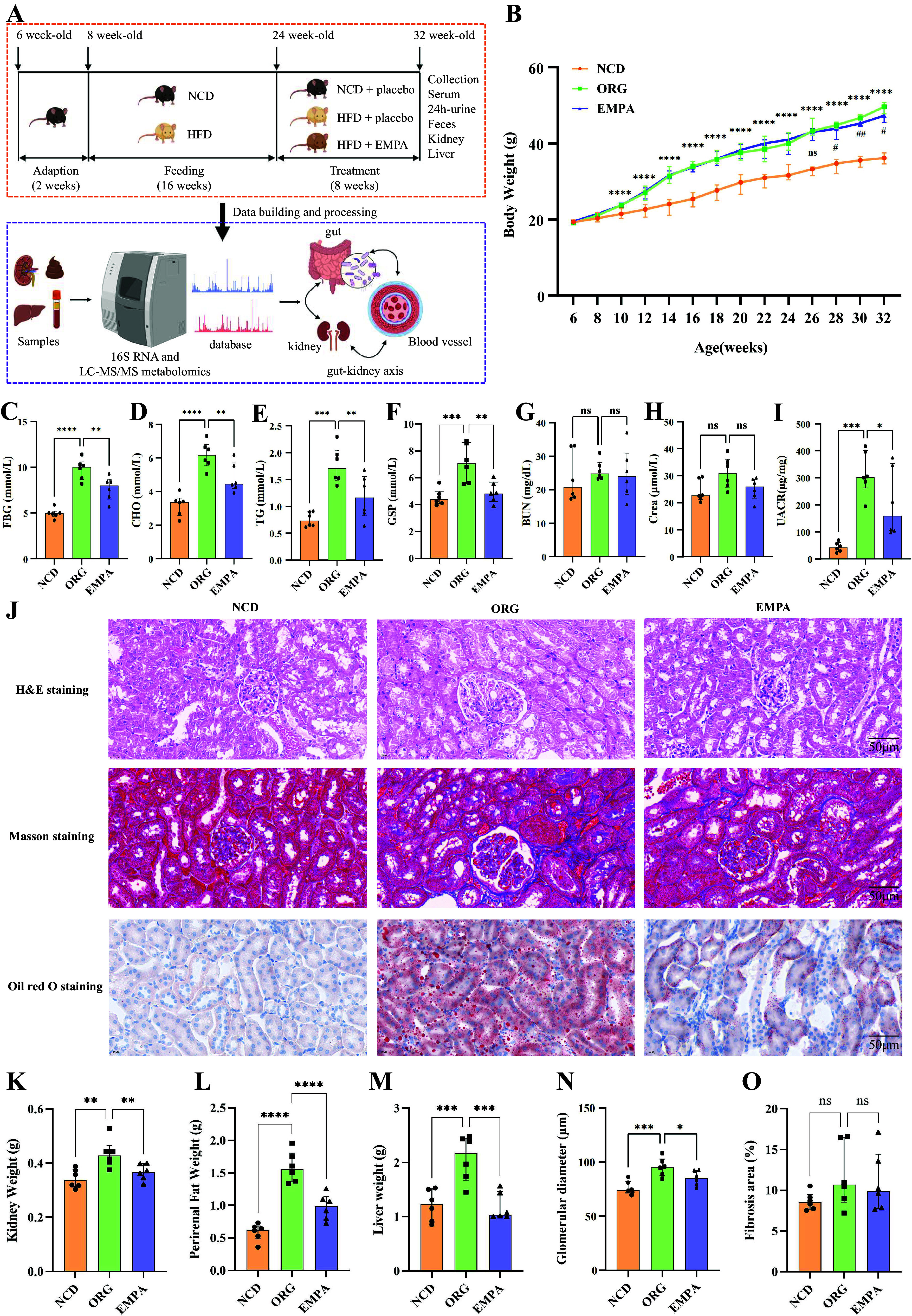
Study design and basic characteristics in obesity-related glomerulopathy (ORG) mice. *A*: the workflow of the animal experiment. *B*: the weekly body weight of mice from 8 to 32 wk. The level of FBG (*C*), CHO (*D*), TG (*E*), GSP (*F*), BUN (*G*), Crea (*H*), and UACR (*I*) in different groups. *J*: renal tissue pathology (×200) of mice in each group (H&E staining, Masson staining, and Oil red O staining). The combined weights of the left and right kidneys (*K*), the combined weights of the left and right perirenal fat (*L*), liver weights (*M*), average glomerular diameter (*N*), and percentage of kidney fibrosis (*O*) in different groups. *P < 0.05; **P < 0.01; ***P < 0.001; ****P < 0.0001 when ORG vs. NCD. #*P* < 0.05; ##*P* < 0.01 when EMPA vs. ORG. Figure created with BioRender.com. BUN, blood urea nitrogen; CHO, cholesterol; Crea, creatinine; FBG, fasting blood glucose; GSP, glycosylated serum protein; H&E, hematoxylin-eosin; ns, not significant; TG, triglyceride; UACR, urinary albumin creatinine ration.

Eighteen male C57BLJ mice (6 wk old) were procured from the Experimental Animal Center of Nanjing University. Following a 2-wk adaptation period, the mice were randomly divided into two groups: the normal chow diet group (NCD; *n* = 6) and the high-fat diet group (HFD; *n* = 12). All mice were housed under specific pathogen-free (SPF) conditions with stringent environmental control (temperature: 22 ± 2°C; humidity: 55 ± 10%; 12-h light/dark cycle) and were provided ad libitum access to food and water. The NCD group received a standard rodent diet throughout the study, whereas the HFD group consumed a diet with a 60 kcal % fat content (Soybean oil 3.2% and Lard 31.6%). Starting from *week 16* of HFD dietary intervention, the HFD group was randomly divided into two subgroups: HFD mice treated with placebo for 8 wk (ORG group, *n* = 6) and treated with EMPA for 8 wk (EMPA group, *n* = 6). For the subsequent 8 wk, mice in the EMPA group received daily oral gavage of EMPA (provided by Shanghai McLean Biochemical Technology Co., Ltd.) at a dose of 10 mg/kg, and mice in the NCD and ORG groups received daily oral administration of 10 mg/kg sterile water using the same method. The intervention continued for 8 wk. Weekly body weight measurements were recorded. After 8 wk of treatment, urine and feces samples were collected from all mice using metabolic cages, promptly frozen in liquid nitrogen, and stored at −80°C. Subsequently, the mice were anesthetized using 2,2,2-tribromoethanol via intraperitoneal injection, and whole blood was collected through cardiac puncture. Blood samples were incubated at room temperature for at least 1 h, followed by centrifugation at 3,000 rpm for 15 min to collect the upper serum layer. Kidney and liver specimens were weighed, with one portion rapidly frozen in liquid nitrogen and the other fixed overnight in 4% paraformaldehyde before being embedded in paraffin. All samples were stored at −80°C and later subjected to LC-MS/MS analysis. The experimental procedures were approved by the Ethics Committee for Animal Experiments (Approval No: 2021070101) and conducted following the National Guidelines for Experimental Animal Welfare. A detailed illustration of the experimental procedures is presented in Fig. 1*A.*

### Biochemical and Histopathological Examination

Serum biochemical analysis was conducted using a fully automatic biochemical analyzer. The serum concentrations of cholesterol (CHO), triglyceride (TG), glycosylated serum protein (GSP), blood urea nitrogen (BUN), and creatinine (Crea) were assayed by an automatic biochemical analyzer (Rayto, Shenzhen, China). Fasting blood glucose (FBG) levels were measured using a glucometer (FreeStyle Freedom Lite, Abbott, UK). Urine creatinine (UCR) levels and urine microalbuminuria albumin levels were determined using the UCR enzyme-linked immunoassay kit (JL51901, J&L Biological, Shanghai, China) and microalbuminuria (MAU/ALB) enzyme-linked immunoassay kit (JL-T-0928, J&L Biological, Shanghai, China), respectively. Kidney samples preserved in paraffin wax were sectioned into 4-µm thin slices for renal histopathological analysis. These slices underwent staining with three different dyes: hematoxylin-eosin (H&E), Masson, and Oil red O. Glomerular size in the kidney filters was assessed by analyzing 10 random areas (25 × 25 µm) in each H&E-stained slice (×200 magnification) using ImageJ software, and the average glomerular diameter was calculated. The extent of fibrotic areas (blue-colored) was determined by examining 10 fields of view in each Masson-stained slice to assess the degree of scarring in the kidney. Lipid accumulation of the kidney was assessed through Oil red O staining (red-stained). Results were reported based on consensus from at least two independent observers, and representative photomicrographs were documented.

### rRNA Gene Sequencing

#### Construct the sequencing library.

We extracted total DNA from the fecal samples. Primers targeting conserved regions within the extracted DNA were designed, followed by polymerase chain reaction (PCR) amplification. The amplified products underwent purification, quantification, normalization, and rigorous quality control. The qualified library was subsequently sequenced on the Illumina NovaSeq 6000 platform.

#### Data preprocessing.

Sequences were filtered and trimmed using Trimmomatic (V 0.33) and Cutadapt (1.9.1) to obtain clean reads. Subsequently, the clean reads from each sample were merged using USEARCH (V10) and length-filtered based on various regions. Finally, the data2 method in QIIME2 (2020.6) was used to denoise the sequences and eliminate chimeric reads, resulting in the generation of high-quality, nonchimeric reads.

#### Amplicon sequence variant analysis.

The preprocessed data underwent amplicon sequence variant (ASV) analysis to identify bacterial strains present in the samples. The sequences were annotated using the Naive Bayes classification method with the SILVA database as a reference, resulting in species classification information for each feature. Subsequently, the community composition of each sample was analyzed at multiple taxonomic levels, including phylum, class, order, family, genus, and species. Species abundance tables for different taxonomic levels were generated using QIIME software, and further analysis was performed using R software (R Foundation for Statistical Computing).

#### Beta diversity analysis.

The nonmetric multidimensional scaling (NMDS) method implemented in the QIIME software was used to evaluate the beta diversity of species within each sample. In addition, permutation analysis of multivariate variance (PERMANOVA) was conducted to determine the significant dissimilarities in beta diversity across the three groups. The analysis was carried out using the vegan package in R, whereas the plots were generated using Python. A larger *R*^2^ value signifies a greater distinction between the groups. The rank sum test was used to assess differences between groups. Line discriminant analysis (LDA) effect size (LEfSe) was used to identify the main distribution characteristics across different groups.

### Metabolomic Analyses

#### Metabolites extraction.

To measure metabolites, 25 mg of each tissue sample (kidney, fecal, and liver) were combined with 500 μL of extraction solvent (methanol:acetonitrile:water in a 2:2:1 ratio, plus an isotopically labeled internal standard mix). The mixture was homogenized at 35 Hz for 4 min and sonicated for 5 min in an ice-water bath, repeating these steps up to three times. For serum samples, we allowed 50 μL to reach room temperature before adding 200 μL of the same extraction solvent into an EP tube. After vortexing for 30 s and sonicating for 5 min, the processed samples were incubated at −40°C for an hour and centrifuged at 12,000 rpm for 15 min at 4°C. The supernatant was collected for analysis, and an aliquot from each sample was reserved for LC-MS/MS quality control.

#### LC-MS/MS analyses.

LC-MS/MS analysis was performed using an ultra-highperformance liquid chromatography (UHPLC) system (Vanquish, Thermo Fisher Scientific) equipped with a UPLC BEH Amide column (2.1 mm × 100 mm, 1.7 μm) and a Q Exactive HFX mass spectrometer (Orbitrap MS, Thermo). The mobile phase composition included 25 mmol/L ammonium acetate and 25 mmol/L ammonia hydroxide in water (pH = 9.75) for mobile phase A, and acetonitrile for mobile phase B. The auto-sampler temperature was maintained at 4°C, with an injection volume of 3 μL. Both MS and MS/MS data were acquired using the QE HFX mass spectrometer controlled by Xcalibur software (Thermo Fisher Scientific). For the electrospray ionization (ESI) source, we set the following conditions: a sheath gas flow of 30 Arb and an auxiliary gas flow of 25 Arb. The capillary was heated to 350°C. Resolutions for full MS and MS/MS were set at 60,000 and 7,500, respectively. In NCE mode, collision energies of 10, 30, and 60 were applied. The spray voltages were adjusted to 3.6 kV in positive mode and −3.2 kV in negative mode.

#### Data processing.

We used ProteoWizard to convert raw data into mzXML format. Peak detection and data processing were conducted using our custom R package, leveraging XCMS for extraction, alignment, and integration. Metabolite annotation involved comparison against our BiotreeDB (V2.1), applying a 0.3 cutoff for annotation. To address missing values, we imputed half the minimum detected value. Given the low incidence of missing values in our dataset, this conservative imputation method avoids the influence of extreme values and preserves the integrity of the data set. Normalization was carried out based on each sample’s total ion intensity (TIC) before proceeding with further analysis.

#### Bioinformatic analyses.

Multivariate statistical analysis included unsupervised principal component analysis (PCA) for observing metabolic profiles, identifying outliers, and assessing QC sample stability. Sparse partial least squares discriminant analysis (sPLS-DA) characterized metabolic feature separation among groups and screened biomarkers. The variable importance in projection (VIP) score identified metabolite contributions, with VIP scores >1, *P* < 0.05 (Student’s *t* test), and |fold change|> 1, indicating significantly differentially expressed metabolites (DEMs). Volcano plots were generated using the R package “ggplot.” Kyoto Encyclopedia of Genes and Genomes (KEGG) enrichment analysis identified potential pathways of metabolites, with the metabolites classified according to the Human Metabolome Database (HMDB).

### MetOrigin Analyses

Preprocessed 16S rRNA gene sequencing and metabolomics data were uploaded to the MetOrigin website (https://metorigin.met-bioinformatics.cn/home/) for correlation analysis. The platform integrated both datasets and computed Spearman’s rank correlation coefficients (r_s_) between the relative abundance of bacterial species and metabolites. Correlation coefficients ranged from −1 to 1, indicating the degree and direction of the relationships between the two variables. To control for multiple testing, a false discovery rate (FDR) adjustment was implemented, and significant correlations were determined based on a predetermined cutoff threshold (e.g., FDR-adjusted *P* < 0.05).

### Statistical Analyses

Biochemical indicators were analyzed using SPSS Statistics v. 25.0 (IBM), GraphPad Prism 9, and R software (R Foundation for Statistical Computing, Vienna, Austria, v3.5.3). One-way ANOVA was used to compare data among the three groups, and Holm–Šídák’s multiple comparisons test was used to calculate statistical differences between each pair of groups. Normally distributed data were expressed as means ± SD, with *P* < 0.05 considered statistically significant.

## RESULTS

### Effects of EMPA on Biochemical Parameters and Renal Histopathology in ORG Mice

The workflow of this study is presented in Fig. 1*A.* The body weight of the mice in the ORG and EMPA groups was significantly higher than that of the NCD group from the 11th wk to the 32th wk (*P* < 0.05; Fig. 1*B*; Supplemental Table S1). Furthermore, from the 24th wk onwards, the body weight of the mice in the ORG group was significantly higher than that of the EMPA treatment group (all *P* < 0.05; Fig. 1*B*; Supplemental Table S1). This aligns with previous studies demonstrating the effectiveness of SGLT2 inhibitors in promoting weight loss and reducing overall fat mass (17, 18, 26). Biochemical analyses revealed that the levels of FBG, CHO, TG, GSP, and urinary albumin creatinine ration (UACR) were significantly increased in ORG mice compared with NCD mice (all *P* < 0.05); EMPA treatment improved these biochemical parameters (all *P* < 0.05) (Fig. 1, *C*–*G* and *I*; Supplemental Table S2). The results align with previous studies, illustrating that EMPA treatment promotes lipid utilization in diet-induced obese mice (19). We observed no significant difference in serum creatinine levels among the three groups (Fig. 1*H*; Supplemental Table S2). The combined weights of the left and right kidneys, the combined weights of the left and right perirenal fat, the ratio of perirenal fat to body weight, and liver weights were significantly increased in ORG mice compared with NCD mice (*P* < 0.05) (Fig. 1, *K–M*; Supplemental Table S3). EMPA treatment mitigated the increase in kidney, perirenal fat, the ratio of perirenal fat to body weight, and liver weights compared with ORG mice (*P* < 0.05; Fig. 1, *K–M*; Supplemental Table S3). Subsequently, we performed renal histopathological of H&E, Masson, and Oil red O staining (Fig. 1*J*). Histopathological examination of ORG mice revealed significant renal changes, including enlarged glomeruli and notable lipid deposition in the renal tissue (Fig. 1*J*). These findings are indicative of renal complications related to obesity. Importantly, no significant renal fibrosis was observed (Fig. 1*O*; Supplemental Table S2), highlighting that the primary pathological features were glomerular enlargement and lipid accumulation. EMPA treatment significantly improved these pathological features by reducing glomerular diameter and ameliorating lipid deposition, effectively normalizing renal structure and histopathology (*P* < 0.001; Fig. 1, *J* and *N*; Supplemental Table S2). These findings highlight the reno-protective effects of EMPA in improving histological parameters in ORG mice.

### Effects of EMPA on the Gut Microbiota Profiles in ORG Mice

**Figure 2.**
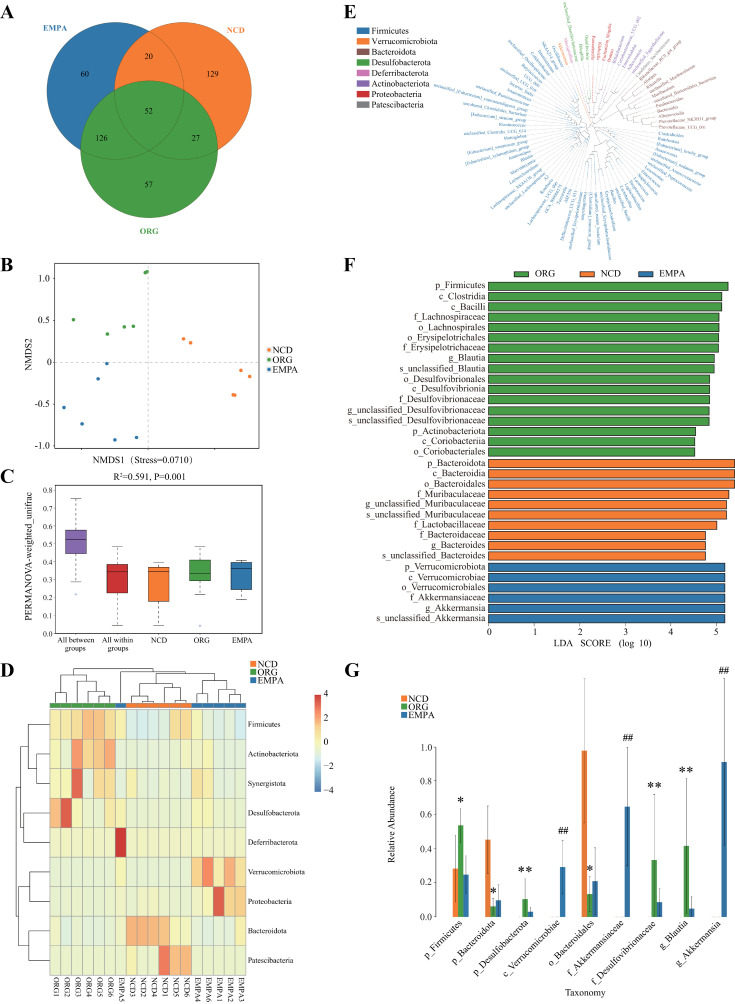
Gut microbiota profiles in obesity-related glomerulopathy (ORG) mice. *A*: the Venn diagram illustrated the common features among the three groups. *B*: nonmetric multidimensional scaling (NMDS) plot showed the gut microbial distribution in different groups. *C*: permutation analysis of multivariate variance (PERMANOVA) analysis evaluated the changes in beta diversity among different groups. *D*: the heatmap showed the relative abundance of different taxa in different samples. *E*: phylogenetic tree at the genus level was provided, where each branch represented a species, and the length represented the evolutionary distance from the phylum level to the genus level. *F*: line discriminant analysis (LDA) distribution histogram at different taxonomic levels. The length of the bar chart represents the effect size of the differential abundance species (LDA score >4.0). *G*: the bar chart of taxonomy illustrated relative abundance of gut microbiota in the three groups. c, class; f, family; g, genus; o, order; p, phylum; s, species. *P < 0.05; **P < 0.01 when ORG vs. normal chow diet (NCD). ##*P* < 0.01 when empagliflozin (EMPA) vs. ORG.

We identified a total of 471 ASVs from feces. Among these, 52 ASVs were common to all groups, and 129, 57, and 60 ASVs were unique to the NCD, ORG, and EMPA groups, respectively (Fig. 2*A*). The relative abundance of microbial taxa was analyzed at six taxonomic levels in each sample (Supplemental Table S4). The NMDS analysis revealed significant variations in beta diversity among the three groups (Fig. 2*B*). The subsequent PERMANOVA analysis further confirmed that the dissimilarities between groups exceeded the dissimilarities within groups (*P* < 0.05; Fig. 2*C*). At the phylum level, NCD mice showed enrichment in *Bacteroidota* and *Patescibacteria*, whereas ORG mice exhibited enrichment in *Firmicutes*, *Actinobacteriota*, *Synergistota*, and *Desulfobacterota* (Fig. 2*D*). EMPA mice displayed an increased abundance of *Verrucomicrobiota* and *Proteobacteria* (Fig. 2*D*). The phylogenetic tree of fecal microbiota (Fig. 2*E*) highlights that the majority at the genus level originates from the phylum *Firmicutes*. LDA distribution histogram illustrated the prevailing microbiota within different groups (Fig. 2*F*). Specifically, in NCD mice, dominant microbial communities were affiliated with the *Bacteroidota* phylum. Conversely, ORG mice displayed dominant communities primarily associated with the *Firmicutes* phylum. In addition, mice treated with EMPA demonstrated a remarkable transition toward the *Verrucomicrobiota* phylum.

The bar chart of microbial taxonomy illustrated the relative abundance of gut microbiota in the three groups (Fig. 2*G*; Supplemental Table S5). In comparison with the NCD group, mice in the ORG group exhibited a significant decrease in the *Bacteroidota* phylum and *Bacteroidales* order, along with a significant increase in the *Firmicutes* phylum, *Desulfobacterota* phylum, *Desulfovibrionaceae* family, and *Blautia* genus. These alterations were partially reversed by EMPA treatment. Notably, the *Verrucomicrobiae* class, *Akkermansiaceae* family, and *Akkermansia* genus (all belonging to the *Verrucomicrobiota* phylum) showed a significant increase in the EMPA group, which were scarcely detected in the NCD and ORG groups. This suggests that EMPA treatment might reshape the gut microbiota, particularly increasing the abundance of the *Verrucomicrobiota* phylum.

### Effects of EMPA on the Renal Metabolism Profiles in ORG Mice

**Figure 3.**
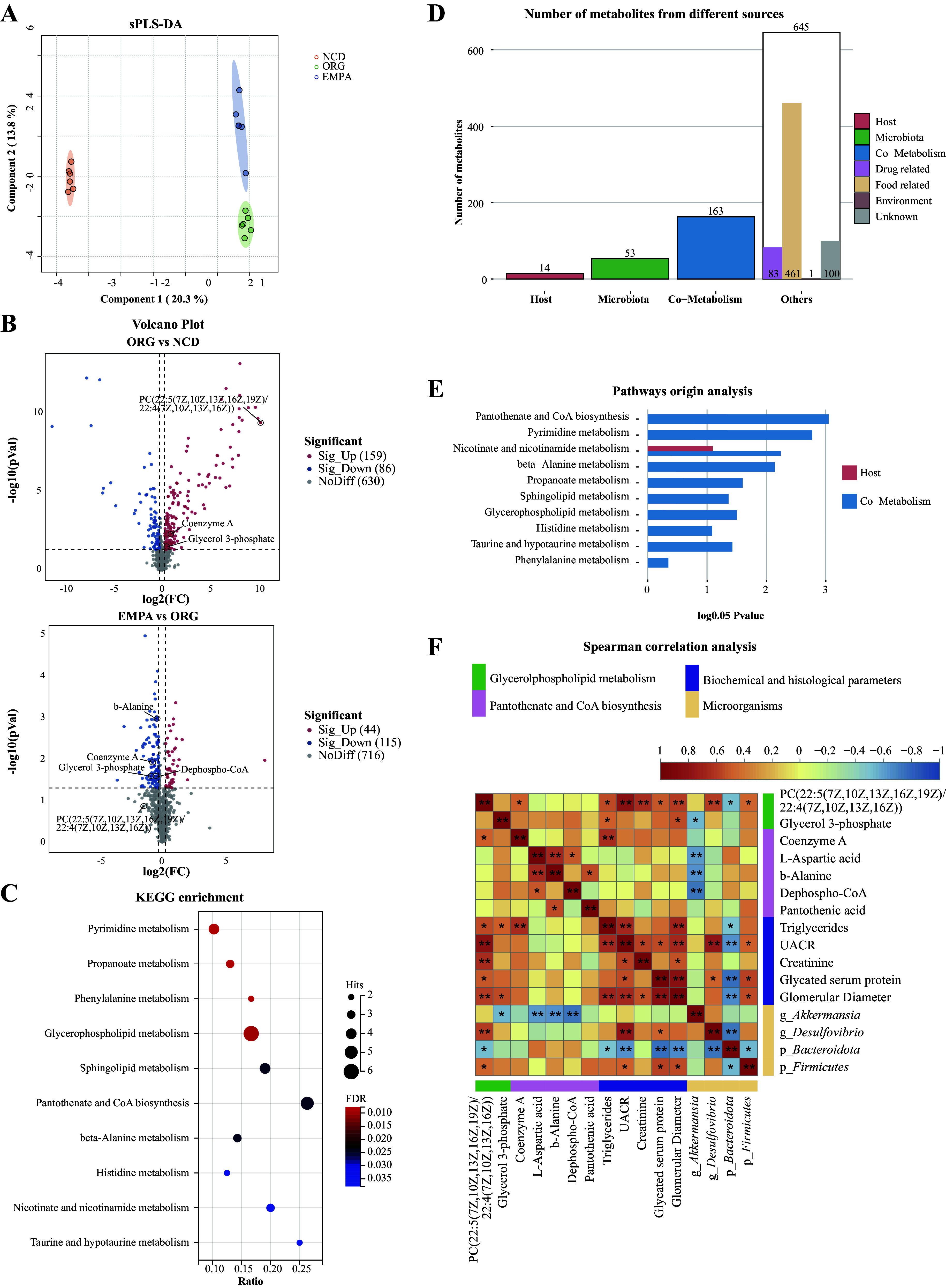
Renal metabolic profiles in obesity-related glomerulopathy (ORG) mice. *A*: the sparse partial least squares discriminant analysis (sPLS-DA) analysis showed renal metabolic profiles in the three groups. *B*: volcano plots visualized the differentially expressed metabolites (DEMs) between ORG vs. normal chow diet (NCD) and empagliflozin (EMPA) vs. ORG. *C*: Kyoto encyclopedia of genes and genomes (KEGG) pathway analysis of differentially expressed metabolites (DEMs) in kidney between the EMPA and ORG groups. *D*: the column graph of MetOrigin analysis showed the originating sources of kidney metabolites. *E*: the bar chart of pathways origin analysis illustrated the *P* value of metabolism pathway derived from the host, gut microbiota, or coaction of both host and gut microbiota. *F*: heatmap of Spearman correlation analysis revealed potential associations between metabolites, biochemical and histological parameters, and gut microbiota in all samples. The green font represented DEMs in the glycerophospholipid metabolism, the pink font represented DEMs in the pantothenate and CoA biosynthesis pathway, the purple font represented biochemical and histological parameters, and the yellow font represented the microorganisms. *P < 0.05; **P < 0.01. g, genus; p, phylum; PC, phosphatidylcholine. g, genus; p, phylum; PC, phosphatidylcholine.

A total of 875 metabolites were identified from the kidney, with 428 successfully matched by comparing the KEGG and HMDB databases (Supplemental Table S6). The sPLS-DA model illustrated distinct metabolic profiles among the three groups (Fig. 3*A*). A total of 245 DEMs were identified between the ORG and NCD groups using the criteria VIP >1, *P* < 0.05, and |FC| >1.0. Among these DEMs, 159 were upregulated and 86 were downregulated. Furthermore, between the EMPA and ORG groups, 159 DEMs were identified, with 44 upregulated and 115 downregulated (Fig. 3*B*). In addition, 39 metabolites were found to be coexpressed in both comparison groups (ORG vs. NCD, EMPA vs. ORG) and were classified as EMPA-altered metabolites (*P* < 0.05; Supplemental Table S6). Importantly, EMPA treatment upregulated reno-protective metabolites such as carnosine (27) and flavone (28), whereas downregulated metabolites associated with obesity, including glycerol 3-phosphate (G3P) (29) and coenzyme A (CoA) (30). These observed modifications in kidney metabolism induced by EMPA prompted an investigation into the specific metabolic pathways in ORG mice. We further analyzed the 428 successfully matched metabolites in the EMPA and ORG groups using MetaboAnalyst 5.0. The analysis unveiled 41 significant enrichment pathways, primarily associated with amino acid, carbohydrate, lipid, cofactor and vitamin, and nucleotide metabolism (*P* < 0.05; Supplemental Table S7). Glycerophospholipid metabolism, pyrimidine metabolism, and pantothenate and CoA metabolism demonstrated the most significant enrichment of metabolites (Fig. 3*C*; Supplemental Table S7). Notably, G3P in glycerophospholipid metabolism and CoA in the pantothenate and CoA biosynthesis pathway were identified as EMPA-altered metabolites (Fig. 3*B*; Supplemental Table S6). This observation highlights a role of EMPA in reprogramming these two pathways in renal metabolism through its impact on G3P and CoA.

MetOrigin analysis was performed to explore the potential role of gut microbiota in the reprogramming of kidney metabolites. This analysis revealed that 53 metabolites originated from gut microbiota, whereas 163 metabolites were derived from both gut microbiota and host (Fig. 3*D*). Pathway origin analysis further suggested that the majority of the enriched pathways were generated through microbiota-host cometabolism (Fig. 3*E*). Spearman correlation analysis was used to explore potential associations among metabolites, biochemical and histological parameters, and gut microbiota in all samples. The results revealed a significant positive correlation between PC [22:5(7z,10z,13z,16z,19z)/22:4(7z,10z,13z,16z)] and the *Firmicutes* phylum (r_s_ = 0.471, *P* = 0.048), as well as with the *Desulfovibrio* genus (r_s_ = 0.594, *P* = 0.009). Furthermore, PC [22:5(7z,10z,13z,16z,19z)/22:4(7z,10z,13z,16z)] exhibited a positive correlation with TG (r_s_ = 0.548, *P* = 0.018), UACR (r_s_ = 0.812, *P* = 0.00004), and glomerular diameters (r_s_ = 0.597, *P* = 0.009) (Fig. 3*F*; Supplemental Table S8). In addition, G3P showed a positive correlation with TG (r_s_ = 0.481, *P* = 0.043) and glomerular diameters (r_s_ = 0.542, *P* = 0.020). CoA showed a positive correlation with TG (r_s_ = 0.649, *P* = 0.0035). These findings suggest a potential involvement of PC [22:5(7z,10z,13z, 16z,19z)/22:4(7z,10z,13z, 16z)], G3P, and CoA in the promotion of lipid synthesis and kidney injury. Interestingly, a significant negative correlation between G3P and the genus *Akkermansia* was observed (r_s_=−0.536, *P* = 0.022) (Fig. 3*F*; Supplemental Table S8), suggesting the beneficial effect of *Akkermansia* on improving renal lipid metabolism.

### Effects of EMPA on the Fecal Metabolism Profiles in ORG Mice

**Figure 4.**
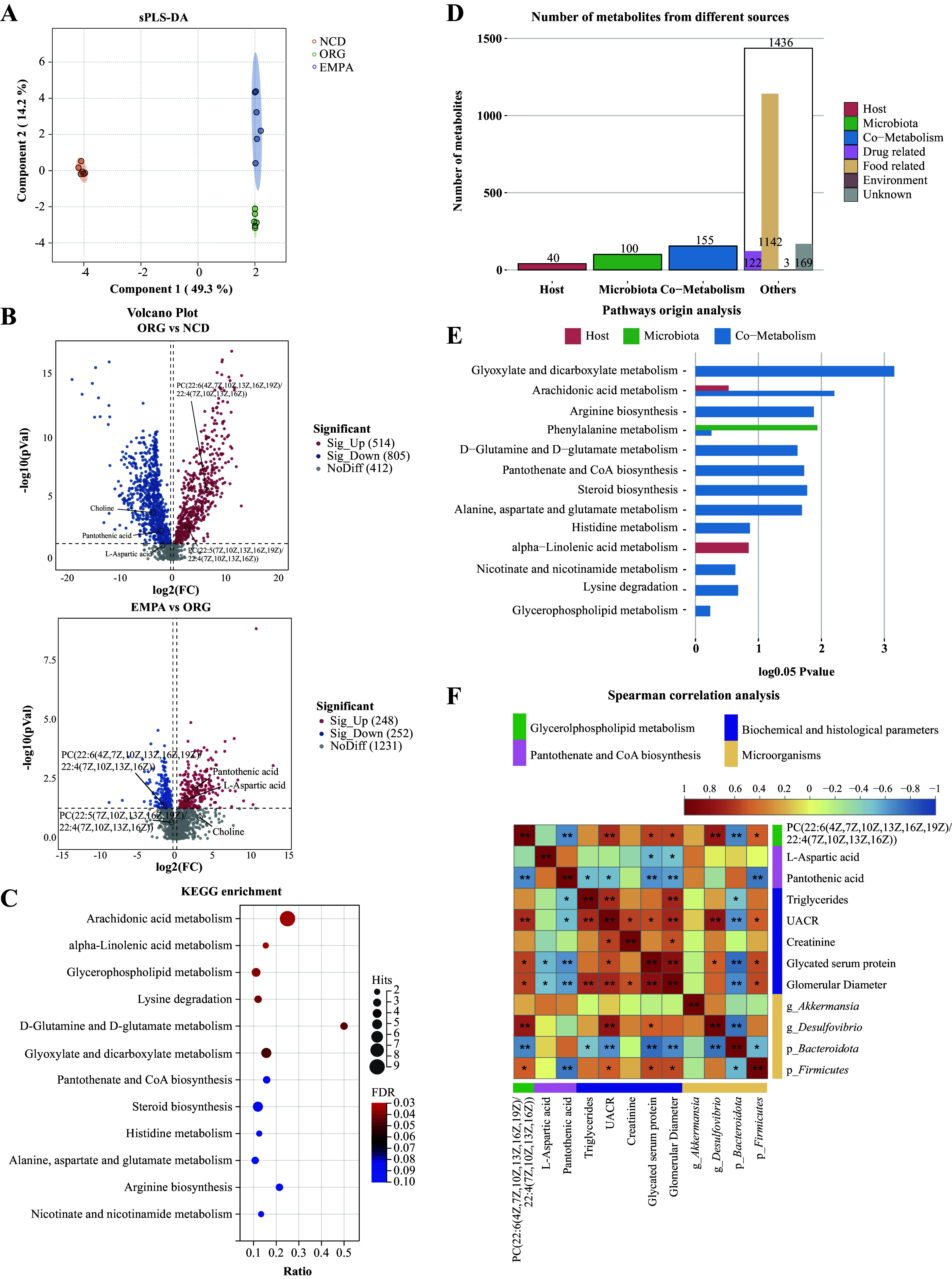
Fecal metabolic profiles in obesity-related glomerulopathy (ORG) mice. *A*: the sparse partial least squares discriminant analysis (sPLS-DA) analysis showed fecal metabolic profiles in the three groups. *B*: volcano plots visualized the differentially expressed metabolites (DEMs) between ORG vs. normal chow diet (NCD) and empagliflozin (EMPA) vs. ORG. *C*: Kyoto encyclopedia of genes and genomes (KEGG) pathway analysis of differentially expressed metabolites (DEMs) in feces between the EMPA and ORG groups. *D*: the column graph of MetOrigin analysis showed the originating sources of feces metabolites. *E*: the bar chart of pathways origin analysis illustrated the *P* value of metabolism pathway derived from the host, gut microbiota, or coaction of both host and gut microbiota. *F*: heatmap of Spearman correlation analysis revealed potential associations between metabolites, biochemical and histological parameters, and gut microbiota in all samples. The green font represented DEMs in the glycerophospholipid metabolism, the pink font represented DEMs in the pantothenate and CoA biosynthesis pathway, the purple font represented biochemical and histological parameters, and the yellow font represented the microorganisms. *P < 0.05; **P < 0.01. g, genus; p, phylum; PC, phosphatidylcholine.

In the feces, a total of 1,731 metabolites were identified, and 603 of them were matched with compounds in the KEGG and HDMB databases (Supplemental Table S9). The sPLS-DA model demonstrated distinct metabolic profiles among the three groups (Fig. 4*A*). Using the criteria VIP >1, *P* <0.05, and |FC| >1.0, 1,319 DEMs were identified between the ORG and NCD groups, with 514 upregulated and 805 downregulated metabolites. In addition, between the EMPA and ORG groups, 500 DEMs were identified, with 248 upregulated and 252 downregulated metabolites (Fig. 4*B*). Among these DEMs, 100 were identified as EMPA-altered metabolites (Supplemental Table S9). To explore the changes in metabolic pathways in ORG mice, 603 matched metabolites from the EMPA and ORG groups underwent KEGG enrichment analysis using MetaboAnalyst 5.0. This analysis revealed 31 significant enrichment pathways, with the top three pathways identified as arachidonic acid metabolism, alpha-linolenic acid metabolism, and glycerophospholipid metabolism (*P* < 0.05, Fig. 4*C*, Supplemental Table S10). Notably, PC [22:6(4Z,7Z,10Z, 13Z,16Z,19Z)/22:4(7Z,10Z,13Z,16Z)] enriched in glycerophospholipid metabolism was found to be the most abundant phosphatidylcholines (PCs) altered by EMPA treatment (Supplemental Table S9). Despite the lack of significance observed in the KEGG enrichment analysis for the pantothenate and CoA biosynthesis pathway (Fig. 4*C*; Supplemental Table S10), the administration of EMPA yielded a significant increase in l-aspartic acid (L-Asp) and pantothenic acid levels (Supplemental Table S9), both of which play a pivotal role in CoA biosynthesis.

MetOrigin analysis was used to explore the potential role of gut microbiota in the reprogramming of feces metabolites. A total of 100 metabolites were identified as microbiota metabolites, whereas 155 metabolites were recognized as microbial-host cometabolites (Fig. 4*D*). The analysis of pathway origin indicated that a significant proportion of the metabolites enriched in the pathways were generated through the process of cometabolism (Fig. 4*E*). Furthermore, the Spearman correlation analysis revealed a significant positive association between the levels of PC [22:6(4Z,7Z,10Z,13Z,16Z,19Z)/22:4(7Z,10Z,13Z,16Z)] and several factors, including UACR (r_s_ = 0.645, *P* = 0.004), GSP (r_s_ = 0.566, *P* = 0.014), as well as the phylum *Firmicutes* (r_s_ = 0.496, *P* = 0.036) and the genus *Desulfovibrio* (r_s_ = 0.746, *P* = 0.0004). Moreover, pantothenic acid exhibited a negative correlation with TG (r_s_=−0.507, *P* = 0.032), UACR (r_s_=−0.509, *P* = 0.031), GSP (r_s_=−0.637, *P* = 0.004), glomerular diameter (r_s_=−0.593, *P* = 0.009), and *Firmicutes* (r_s_=−0.707, *P* = 0.001) (Fig. 4*F*; Supplemental Table S11). The results suggest that EMPA treatment resulted in a downregulation in the levels of PC [22:6(4Z,7Z,10Z, 13Z,16Z,19Z)/22:4(7Z,10Z, 13Z,16Z)] in feces, accompanied by an upregulation in the concentrations of pantothenic acid. Moreover, these changes appear to be associated with alterations in the abundance of the phylum *Firmicutes*.

### Effects of EMPA on the Liver Metabolism Profiles in ORG Mice

**Figure 5.**
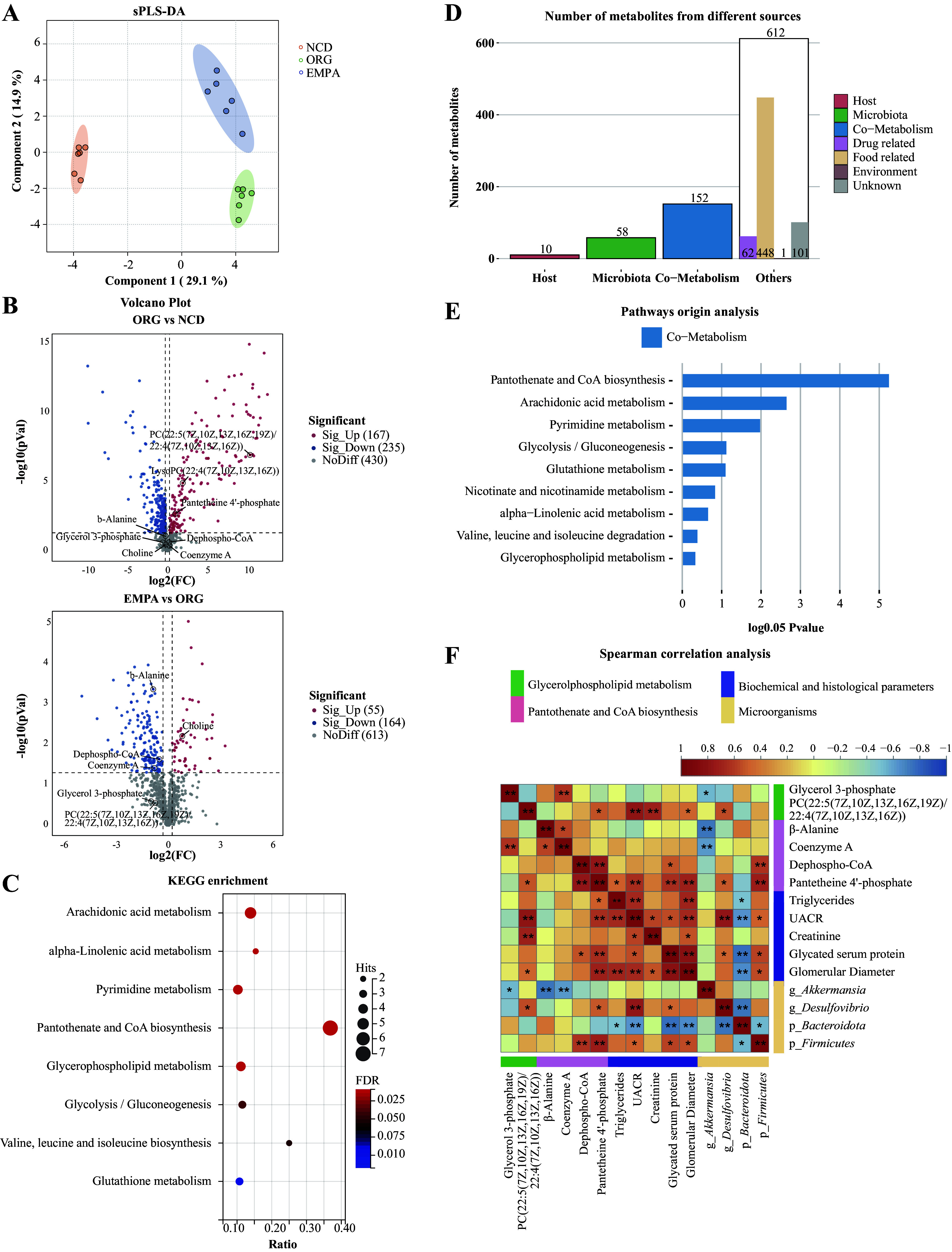
Liver metabolic profiles in obesity-related glomerulopathy (ORG) mice. *A*: the sparse partial least squares discriminant analysis (sPLS-DA) analysis showed liver metabolic profiles in the three groups. *B*: Volcano plots visualized the differentially expressed metabolites (DEMs) between ORG vs. normal chow diet (NCD) and empagliflozin (EMPA) vs. ORG. *C*: Kyoto encyclopedia of genes and genomes (KEGG) pathway analysis of DEMs in liver between the EMPA and ORG groups. *D*: the column graph of MetOrigin analysis showed the originating sources of liver metabolites. *E*: the bar chart of pathways origin analysis illustrated the *P* value of metabolism pathway derived from the host, gut microbiota, or coaction of both host and gut microbiota. *F*: heatmap of Spearman correlation analysis revealed potential associations between metabolites, biochemical and histological parameters, and gut microbiota in all samples. The green font represented DEMs in the glycerophospholipid metabolism, the pink font represented DEMs in the pantothenate and CoA biosynthesis pathway, the purple font represented biochemical and histological parameters, and the yellow font represented the microorganisms. *P < 0.05; **P < 0.01. g, genus; p, phylum; PC, phosphatidylcholine.

In the liver, a total of 832 metabolites were identified, and 603 of them were matched with compounds in the KEGG and HDMB databases (Supplemental Table S12). We used the sPLS-DA model to delineate distinctive metabolic profiles in the liver (Fig. 5*A*). Using the criteria VIP >1, *P* <0.05, and |FC| >1.0, we identified 402 DEMs between the ORG and NCD groups, with 167 upregulated and 235 downregulated metabolites. In addition, we identified 219 DEMs between the EMPA and ORG groups, with 55 upregulated and 164 downregulated metabolites (Fig. 5*B*). Subsequently, we analyzed the 397 metabolites obtained from the EMPA and ORG groups using MetaboAnalyst 5.0. Our findings revealed 39 significant enrichment pathways, with the most prominent pathways being pantothenate and CoA biosynthesis, glycerophospholipid metabolism, pyrimidine metabolism, and arachidonic acid metabolism (*P* < 0.05, Fig. 5*C*; Supplemental Table S13). In contrast to the metabolic pathway analysis findings from the kidney, no significant changes in the levels of G3P were observed in the liver. However, in the EMPA treatment group, a significant reduction was observed in the levels of β-alanine, dephospho-CoA, pantetheine 4′-phosphate, and CoA in the liver, all of which are involved in the biosynthesis of pantothenate and CoA (Supplemental Table S12). This observation suggests that EMPA might impact the biosynthesis of pantothenate and CoA in the liver.

We used MetOrigin analysis to explore the potential role of gut microbiota in the reprogramming of liver metabolites and identified 58 metabolites originating from microbiota and 152 metabolites derived from both gut microbiota and host (Fig. 5*D*). In addition, the pathway analysis indicated that a significant portion of the enriched metabolites in the pathway were generated through microbial-host cometabolism (Fig. 5*E*). The subsequent Spearman analysis revealed no significant correlation between G3P, CoA, β-alanine, and dephospho-CoA with renal injury parameters. Nevertheless, it was observed that *Akkermansia* exhibited a significant negative association with G3P (r_s_=−0.535, *P* = 0.022) and CoA (r_s_=−0.605, *P* = 0.008) (Fig. 5*F*; Supplemental Table S14). This finding implies that although hepatic metabolic alterations are not directly associated with renal impairment, they can be modulated by EMPA and the gut microbiota.

### Effects of EMPA on the Serum Metabolism Profiles in ORG Mice

**Figure 6.**
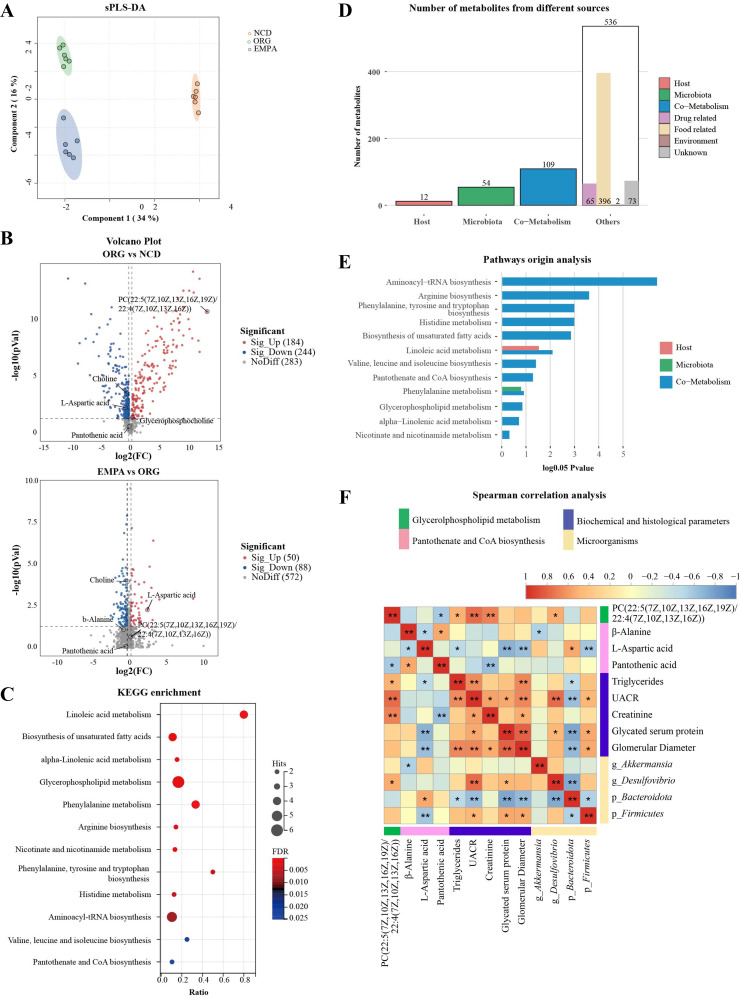
Serum metabolic profiles in obesity-related glomerulopathy (ORG) mice. *A*: the sparse partial least squares discriminant analysis (sPLS-DA) analysis showed serum metabolic profiles in the three groups. *B*: volcano plots visualized the differentially expressed metabolites (DEMs) between ORG vs. normal chow diet (NCD) and empagliflozin (EMPA) vs. ORG. *C*: Kyoto encyclopedia of genes and genomes (KEGG) pathway analysis of DEMs in serum between the EMPA and ORG groups. *D*: the column graph of MetOrigin analysis showed the originating sources of serum metabolites. *E*: the bar chart of pathways origin analysis illustrated the *P* value of metabolism pathway derived from the host, gut microbiota, or coaction of both host and gut microbiota. *F*: heatmap of Spearman correlation analysis revealed potential associations between metabolites, biochemical and histological parameters, and gut microbiota in all samples. The green font represented DEMs in the glycerophospholipid metabolism, the pink font represented DEMs in the pantothenate and CoA biosynthesis pathway, the purple font represented biochemical and histological parameters, and the yellow font represented the microorganisms. *P < 0.05; **P < 0.01. PC, phosphatidylcholine; g, genus; p, phylum.

A total of 711 metabolites were identified from serum, with 330 metabolites successfully matched by comparing the KEGG and HMDB databases (Supplemental Table S15). The application of the sPLS-DA model revealed distinct metabolic profiles among the three groups (Fig. 6*A*). By applying the screening criteria of VIP >1, *P* <0.05, and |FC| >1.0, 428 DEMs were identified between the ORG and NCD groups, consisting of 184 upregulated and 244 downregulated metabolites. In addition, 138 DEMs were identified between the EMPA and ORG groups, with 50 upregulated and 88 downregulated DEMs (Fig. 6*B*). MetaboAnalyst 5.0 was used to analyze the 330 metabolites found in the EMPA and ORG groups. The analysis unveiled 42 significant enrichment pathways (FDR < 0.05; Supplemental Table S16). The top four pathways were linoleic acid metabolism, glycerophospholipid metabolism, phenylalanine metabolism, and tryptophan metabolism (Fig. 6*C*; Supplemental Table S16). Despite the absence of evidence indicating that EMPA treatment alters serum metabolites associated with glycerophospholipid metabolism, a notable increase in serum L-Asp was observed following EMPA treatment (*P* < 0.05; Supplemental Table S15).

MetOrigin analysis was conducted to ascertain the source of metabolites in the EMPA and ORG groups. A total of 54 metabolites were identified as microbiota metabolites, whereas 109 metabolites were recognized as microbial-host cometabolites (Fig. 6*D*). The analysis of pathway origin indicated that the majority of enriched metabolites within the pathway were generated through microbial-host cometabolism (Fig. 6*E*). We conducted a Spearman correlation analysis to investigate the relationship between serum metabolites, renal function, and the gut microbiota. The results revealed that serum PC [22:5(7z,10z,13z,16z,19z)/22:4(7z,10z,13z,16z)] exhibited a positive correlation with TG (r_s_ = 0.505, *P* = 0.033), UACR (r_s_ = 0.761, *P* = 0.0002), and Crea (r_s_ = 0.688, *P* = 0.0016), as well as genus *Desulfovibrio* (r_s_ = 0.530, *P* = 0.024). Conversely, L-Asp demonstrated a negative correlation with TG (r_s_ = −0.477, *P* = 0.045), GSP (r_s_ = −0.680, *P* = 0.002), and glomerular diameters (r_s_ = −0.622, *P* = 0.006), as well as the phylum *Firmicutes* (r_s_ = −0.591, *P* = 0.010) (Fig. 6*E*; Supplemental Table S17).

### Renoprotective Effects of EMPA in ORG Mice through the Regulation of the Gut-Kidney Axis

**Figure 7.**
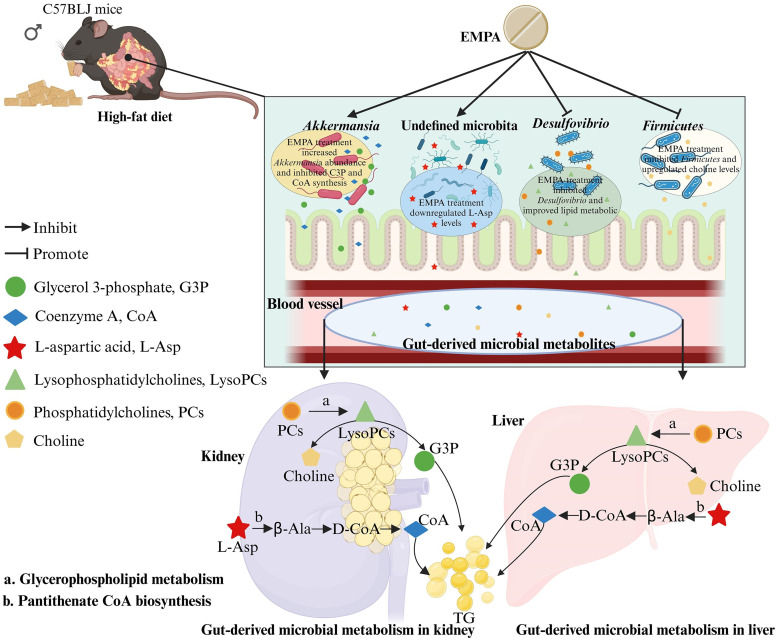
The potential mechanisms of empagliflozin’s (EMPA’s) renoprotective effects in obesity-related glomerulopathy (ORG) mice through the regulation of gut-kidney axis. Lipid metabolic reprogramming associated with ORG and EMPA treatment was identified, specifically involving glycerol 3-phosphate in glycerophospholipid metabolism and coenzyme A in pantothenate and CoA biosynthesis, both of which were upregulated in ORG mice, and EMPA therapy remarkably altered the metabolic process. In addition, MetOrigin analysis revealed a close connection between these EMPA-related lipid metabolic pathways and changes in gut microbiota, marked by decreased abundances of *Firmicutes* and *Desulfovibrio*, and increased abundance of *Akkermansia*. Figure created with BioRender.com. β-Ala, β-alanine; CoA, coenzyme A; G3P, glycerol 3-phosphate; L-Asp, L-aspartic acid; LysoPCs, lysophosphatidyl-cholines; PCs, phosphatidylcholines; TG, triglycerides.

We identified significant metabolic alterations in ORG mice associated with the gut microbiota, predominantly affecting glycerophospholipid metabolism and pantothenate and CoA biosynthesis. Elevated levels of PCs, LysoPCs, and G3P were observed in the feces, kidneys, liver, and serum of ORG mice, with G3P upregulated in the kidneys and livers. Although choline was not detected in the kidneys, a decrease in choline was observed in feces, liver, and serum from ORG mice. EMPA treatment decreased the levels of G3P in the kidney, accompanied by increased levels of choline in the feces and serum (Supplemental Tables S9 and S15). The CoA levels were upregulated in the kidneys of ORG mice, indicating an activation of the pantothenate and CoA biosynthesis pathway (Supplemental Table S6). EMPA treatment downregulated the levels of L-Asp, β-alanine, pantetheine 4′-phosphate, and dephospho-CoA in kidneys and livers, which was associated with reduced levels of CoA in kidneys and livers. In addition, MetOrigin analysis revealed a close connection between these EMPA-related lipid metabolic pathways and changes in gut microbiota, marked by decreased abundances of *Firmicutes* and *Desulfovibrio*, and an increased abundance of *Akkermansia.* Our findings suggest that renoprotective effects of EMPA in ORG mice might be elicited through the regulation of the gut-kidney axis (Fig. 7).

## DISCUSSION

In this study, we performed 16S rRNA sequencing of feces, along with LC-MS/MS metabolomic analysis in kidney, feces, liver, and serum samples from ORG mice. Multitissue metabolomics analysis unveiled metabolic reprogramming in ORG mice, with EMPA treatment inducing alterations in the metabolic profiles. Three EMPA-related lipid metabolic pathways were identified. Specifically, implicating G3P in glycerophospholipid metabolism and CoA in pantothenate and CoA biosynthesis were downregulated after EMPA treatment. Moreover, MetOrigin analysis revealed a strong connection between EMPA-related lipid metabolic pathways and alterations in gut microbiota. This association was characterized by reduced abundances of *Firmicutes* and *Desulfovibrio*, along with an increased abundance of *Akkermansia*.

Alterations in glycerophospholipid metabolism in obesity contribute to insulin resistance and impaired glucose tolerance (31). Elevated levels of PCs, LysoPCs, and nonesterified fatty acids (NEFAs) in the plasma of individuals with obesity positively correlate with the onset of lipotoxicity (32, 33). In addition, elevated glycerophospholipids are associated with the development of CKD (34), and increased PCs are linked to a higher risk of all-cause mortality (35). In our study, altered levels of a broad spectrum of PCs were observed in investigated biological materials (kidney, feces, liver, and serum), consistent with previous research (32, 33). We observed elevated levels of PC [22:5(7Z,10Z, 13Z,16Z,19Z)/22:4(7Z,10Z,13Z,16Z)] in the kidney, serum, and liver samples of ORG mice. Interestingly, the fecal samples exhibited the highest content of PCs, specifically PC (22:6(4Z,7Z,10Z,13Z,16Z,19Z)/22:4 (7Z,10Z,13Z,16Z)). This finding is noteworthy in the context of glycerophospholipid metabolism, where PCs are transformed into LysoPCs by phospholipase A2 (PLA2), ultimately leading to the generation of glycerophosphocholines (GPCs). The kidneys of ORG mice demonstrated elevated G3P levels, indicating a disruption in glycerophospholipid metabolism within the renal tissue of ORG mice. Given that G3P is a vital substrate for TG synthesis in mammalian cells (36), we observed that G3P positively correlated with TG, urinary UACR, and glomerular diameter in Spearman analysis. This finding offers further insights into the potential role of G3P in promoting dyslipidemia and kidney injury. Similar to G3P, the metabolite CoA showed a specific elevation in the kidneys of ORG mice. The derivatives of CoA, namely acetyl-CoA and malonyl-CoA, play pivotal roles in energy and lipid metabolism (37–39). These molecules are crucial for the biosynthesis of fatty acids and the inhibition of mitochondrial fatty acid β-oxidation, thereby promoting triglyceride synthesis in the context of obesity (40). Elevated levels of acetyl-CoA in patients with CKD suggest the inhibition of mitochondrial fatty acid β-oxidation, contributing to renal dysfunction (41). In addition, CoA was identified to play a role in the process of tumorigenesis (42, 43). In our study, Spearman analysis revealed a positive correlation between CoA and TG, as well as UACR in the kidneys of ORG mice. This finding highlights a potential role of CoA in contributing to renal damage in ORG mice. Furthermore, we identified several key intermediate metabolites involved in CoA biosynthesis in the kidney, such as L-Asp, β-alanine, and dephospho-CoA. We observed a significant decrease in the levels of these metabolites in mice treated with EMPA.

It is widely recognized that EMPA improves renal function by reducing glomerular pressure, hyperfiltration, as well as inflammation and fibrosis in the kidneys (44, 45). Consistent with previous studies, our study found that EMPA treatment effectively reduced UACR and glomerular hypertrophy in ORG mice. Beyond their glucose-lowering and blood pressure-reducing capabilities, SGLT2 inhibitors have recently gained attention for their ability to modulate systemic metabolism. Studies have shown that SGLT2 inhibitors can influence lipid metabolism by reducing levels of TG and very low-density lipoprotein (VLDL) through increased lipoprotein lipase activity (46). Simultaneously, SGLT2 inhibitors elevate high-density lipoprotein (HDL) levels via upregulated expression of ATP-binding cassette transporter A1 (ABCA1) and enhanced lecithin-cholesterol acyltransferase (LCAT) activity (46, 47). Osataphan et al. (48) demonstrated that canagliflozin initiates a fasting-like transcriptional and metabolic paradigm, resulting in elevated plasma ketones, reduced adiposity, and improved glucose tolerance and plasma lipid profiles. Our results revealed decreased serum TG levels and decreased lipid accumulation in the renal tissues of mice treated with EMPA, which lends further support to this observation. Moreover, our recent study provided evidence that EMPA induces renal metabolic reprogramming in DKD mice (25). In the current study, the downregulation of G3P and CoA following EMPA treatment provided compelling evidence supporting the role of EMPA in the reprogramming of lipid metabolism. We observed substantial alterations in metabolic profiles of EMPA-altered metabolites across the gut, serum, liver, and kidney. The most notably impacted pathways were glycerophospholipid metabolism and pantothenate and CoA biosynthesis. These findings suggest a potential mechanism for the renoprotective effects of EMPA in ORG mice through the regulation of the gut-kidney axis.

Research indicated that SGLT2 inhibitors modify gut microbial metabolism by promoting glucose delivery into the colon. This process suppresses protein fermentation and reduces the generation of uremic toxins, such as phenols and indoles (49). Kusunoki et al. (50) observed a significant increase in the prevalence of *Ruminococci* following treatment with luseogliflozin or dapagliflozin, thereby promoting the production of short-chain fatty acids (SCFAs). A subsequent study demonstrated that EMPA treatment increased the abundance of *Roseburia*, *Eubacterium*, and *Faecalibacterium*, whereas reducing the abundance of *Escherichia-Shigella*, *Bilophila*, and *Hungatella*. These modifications induced anti-inflammatory and anti-oxidative effects attributed to EMPA, potentially leading to improved insulin sensitivity, enhanced glucose metabolism, and better endothelial function (51). Our findings revealed that EMPA treatment decreased the abundance of the phylum *Firmicutes* and genus *Desulfovibrio*, whereas increasing the abundance of the genus *Akkermansia*. Multiple studies have demonstrated that the consumption of unsaturated fats increases the population of *Firmicutes*, as well as the *Firmicutes*-to-*Bacteroidetes* ratio (52–54). The overgrowth of *Firmicutes* promotes the production and accumulation of uremic toxins, such as indoxyl sulfate, p-cresylsulfate, trimethylamine N-oxide, and phenylacetylglutamine (55–57). These toxins contribute to a variety of detrimental effects, including oxidative stress, inflammation, fibrosis, endothelial dysfunction, and vascular calcification (58, 59). Consistent with previous research, our study observed a significant elevation in the *Firmicutes*-to-*Bacteroidetes* ratio in ORG mice. Spearman analysis further unveiled a positive correlation between the abundance of *Firmicutes* and parameters indicating metabolic disorder (such as TG and GSP), as well as renal damage indices (including Crea, UACR, and glomerular diameter). In addition, our findings indicated a notable increase in the population of *Desulfovibrio* in ORG mice. Previous studies have established that *Desulfovibrio* produces a choline-converting enzyme (Cutc), which transforms choline into trimethylamine N-oxide, thereby augmenting metabolic disorders and renal injury (60, 61). The decreased choline concentrations in the feces and serum of ORG mice suggest a potential influence of *Desulfovibrio* on metabolic disorders (Supplemental Tables S9 and S15). These findings underscore the detrimental impact of *Firmicutes* and *Desulfovibrio* proliferation in ORG mice.

*Akkermansia*, a probiotic recognized for its production of SCFAs, has been demonstrated to be beneficial for individuals with obesity. The absence of *Akkermansia muciniphila* serves as a critical indicator of obesity in both human and mouse models, induced by either a HFD or genetic knockout (62, 63). Studies indicated that supplementation with *Akkermansia muciniphila* has positive effects on various metabolic parameters, including improved insulin sensitivity, reduced insulin levels, and decreased plasma total cholesterol (64). Furthermore, *Akkermansia* modulates the host immune system and protects against intestinal inflammation by synthesizing phosphatidylethanolamine. Phosphatidylethanolamine interacts with Toll-like receptor 2 (TLR2) on intestinal epithelial cells, thereby activating an anti-inflammatory signaling pathway (65). Our results revealed a negative correlation between the abundance of *Akkermansia* and the levels of G3P and CoA, suggesting a potential beneficial role of *Akkermansia* in lipid metabolism. Our findings demonstrated a strong correlation between gut microbiota and metabolites involved in glycerophospholipid metabolism and CoA biosynthesis, suggesting that renoprotective effects of EMPA in ORG mice might be mediated by a modulation of the gut-kidney axis.

To the best of our knowledge, this study represents the first exploration of the potential impact of EMPA on the gut-kidney axis in ORG. We focused on multitissue microbiota profiles, including kidney, feces, liver, and serum, and conducted 16S rRNA gene sequencing of feces to explore the relationship between metabolic alterations and gut microbiota in ORG mice. Simultaneously, we assessed the pivotal role of EMPA in mediating these processes. Nevertheless, we recognize certain limitations. Given the limited sample size in this study, which may constrain the generalizability of the findings, further in-depth investigations are required to elucidate the underlying relationship between gut microbiota and metabolic processes. An additional limitation of our study is the lack of analysis of ketosis. Ketone bodies, such as beta-hydroxybutyrate, acetoacetate, and acetone, play a crucial role in energy metabolism and affect the microbiome. Recent research suggests that that ketogenic diets reduce the phylum *Firmicutes* and genus *Desulfovibrio*, along with increasing *Akkermansia* (66–68) which is consistent with what is observed in our study. As said, we did not measure ketone body levels, which limits our understanding of the full metabolic impact of Empagliflozin on the gut-kidney axis. Future studies should include comprehensive analyses of ketosis to elucidate the potential role of ketone bodies in altering the microbiome and mediating the reno-protective effects observed in obesity-related kidney disease.

In conclusion, this study demonstrated that EMPA treatment significantly improved kidney function and morphology in ORG mice. Results of the study further highlighted that the observed beneficial renal effects might be mediated by modulation of the gut-kidney axis. The potential mechanisms underlying these effects might be associated with the reprogramming of lipid metabolism, specifically involving modifications in glycerophospholipid metabolism, as well as pantothenate and CoA biosynthesis pathways. Moreover, EMPA-mediated modulation of the gut microbiota, particularly *Akkermansia*, seems to play a pivotal role in regulating lipid metabolism by suppressing the synthesis of G3P and CoA. This further highlighted the intricate interactions between gut microbiota and host metabolic processes. Collectively, this study shed light on the potential renoprotective effects of EMPA treatment in ORG mice through modulation of the gut-kidney axis and provided novel treatment strategies to delay the progression of ORG.

## DATA AVAILABILITY

Data will be made available upon reasonable request.

## SUPPLEMENTAL MATERIAL

10.5281/zenodo.13300723 Supplemental Tables S1–S17: https://doi.org/10.5281/zenodo.13300723.

## GRANTS

This work was supported by the National Natural Science Foundation of China (Grant No. 82100716 and 82170690), Guangdong Basic and Applied Basic Research Foundation (Grant No. 2020A1515111209 and 2022A1515110267), The Shenzhen Science and Technology Innovation Committee of Guangdong Province of China (Grant No. JCYJ20210324123200003).

## DISCLOSURES

No conflicts of interest, financial or otherwise, are declared by the authors.

## AUTHOR CONTRIBUTIONS

Z.-H.Z., B.H. and Y.-P.L. conceived and designed research; L.L., T.Z., T.-J.C., X.C., X.-M.Z., K.-W.C., and Z.-Y.D. performed experiments; L.L., T.Z., and T.-J.C. analyzed data; X.-H.W., C.T., L.L., and C.R. interpreted results of experiments; L.L., T.Z., and T.-J.C. drafted manuscript; Y.L., J.-G.H., X.C., B.H., and Y.-P.L. edited and revised manuscript; T.Z., T.-J.C., Y.L., J.-G.H., X.C., X.-M.Z., K.-W.C., Z.-Y.D., L.L., X.-H.W., L.L., C.R., Z.-H.Z., B.H., and Y.-P.L. approved final version of manuscript.
